# Supplementary material for: Angelica sinensis polysaccharide as potential protectants against recurrent spontaneous abortion: focus on autophagy regulation
Source: Front Med (Lausanne). 2025 Jan 15;12:1522503. doi: 10.3389/fmed.2025.1522503 (PMC11774876; doi:10.3389/fmed.2025.1522503)
Supplement: Supplementary material S1 — The main instruments used during the LC-MS process, along with their models/specifications and manufacturers. [file Supplementary_file_1.zip › Metabolomics sequencing data FC1.2/8.代谢通路分析/Exposure_Ctrl/all/KEGG/mmu00030.html]

KEGG PATHWAY: Pentose phosphate pathway - Mus musculus (house mouse)


# Pentose phosphate pathway - Mus musculus (house mouse)


[
Pathway menu
| Organism menu
| Pathway entry
| Show description
| Download
| Help
]

The pentose phosphate pathway is a process of glucose turnover that produces NADPH as reducing equivalents and pentoses as essential parts of nucleotides. There are two different phases in the pathway. One is irreversible oxidative phase in which glucose-6P is converted to ribulose-5P by oxidative decarboxylation, and NADPH is generated [MD:M00006]. The other is reversible non-oxidative phase in which phosphorylated sugars are interconverted to generate xylulose-5P, ribulose-5P, and ribose-5P [MD:M00007]. Phosphoribosyl pyrophosphate (PRPP) formed from ribose-5P [MD:M00005] is an activated compound used in the biosynthesis of histidine and purine/pyrimidine nucleotides. This pathway map also shows the Entner-Doudoroff pathway where 6-P-gluconate is dehydrated and then cleaved into pyruvate and glyceraldehyde-3P [MD:M00008].


##### Option

Scale:


100%

Image resolution:


 High

##### Search

##### ID search

##### Color

##### Module

Complete only
Including 1 block missing
Including any incomplete

- Pathway modules
  - Carbohydrate metabolism
    - Central carbohydrate metabolism
      - M00004
        Pentose phosphate pathway (Pentose phosphate cycle)
      - M00006
        Pentose phosphate pathway, oxidative phase
      - M00007
        Pentose phosphate pathway, non-oxidative phase
      - M00580
        Pentose phosphate pathway, archaea
      - M00005
        PRPP biosynthesis
      - M00008
        Entner-Doudoroff pathway
      - M00308
        Semi-phosphorylative Entner-Doudoroff pathway
      - M00633
        Semi-phosphorylative Entner-Doudoroff pathway
      - M00309
        Non-phosphorylative Entner-Doudoroff pathway
    - Other carbohydrate metabolism
      - M00631
        D-Galacturonate degradation (bacteria)
      - M00061
        D-Glucuronate degradation
      - M00968
        Pentose bisphosphate pathway (nucleoside degradation), archaea
  - Energy metabolism
    - Methane metabolism
      - M00345
        Formaldehyde assimilation, ribulose monophosphate pathway


KGML

Image (png) file 1x
